# Prevention of Biliary Duct Injury in Laparoscopic Cholecystectomy Using Optical Fiber Illumination in Common Bile Duct

**DOI:** 10.4021/gr249e

**Published:** 2010-09-20

**Authors:** Zhi Yong Wang, Fang Xu, Ya Dong Liu, Cheng Gang Xu, Jiang Liang Wu

**Affiliations:** a Department of Digestive Diseases, Affiliated Hospital of Hangzhou Normal University (the Second People’s Hospital of Hangzhou City), Hangzhou, Zhejiang, China; b Department of General Surgery, Affiliated Hospital of Hangzhou Normal University (the Second People’s Hospital of Hangzhou City), Hangzhou, Zhejiang, China

**Keywords:** Laparoscopy, Cholecystectomy, Complication, Biliary duct injury, Cold light, Optical fiber

## Abstract

**Background:**

Biliary duct injury (BDI) is one of the most common complications in laparoscopic cholectecystomy (LC), in this study, we have tried to place an illuminating optical fiber via endoscopy in the CBD during LC, the biliary duct anatomy can be clearly delineated, thus CBD injury is avoided.

**Methods:**

Sixteen patients with chronic cholecystitis or/and cholelithiasis from February 2007 to June 2008 were performed LC with placement of optical fiber in CBD, the fiber with cold light illuminates the whole extrahepatic biliary system. Three 6-mm titanium clips were applied to the soft tissue surrounding the hepatic duct, CBD and the cystic duct confluence with CBD, respectively; one titanium clip was applied to the surface of cystic duct near the infundibulum of gallbladder. The cytic duct, CBD and common hepatic duct were clearly identified and delineated in the operating field and LC was performed.

**Results:**

All the 16 patients were performed LC using this procedure successfully, there were no LC-related complications, nor complications related to endoscopic retrograde cholangiopanceatography (ERCP).

**Conclusions:**

The endoscopically placed optical fiber in the CBD can clearly identify the CBD, Calot’s triangle and the common hepatic duct, this can reduce the bile duct injury in LC and imporve the safety of LC.

## Introduction

Compared with open cholecystectomy, the laparoscopic cholecystectomy (LC) has advantages of less traumatic and rapid recovery, however, intra- or post-operative complications may occur related to LC, these include biliary duct injury (BDI) which is associated with significant perioperative morbidity and mortality [1, 2, 3]; postoperative bile duct remnant stones. In the early years when the LC was first introduced, the incidence of BDI related with LC was approximately three times higher than that of open cholecystectomy [4, 5]. Although various approaches have been developed for prevention of BDI [6, 7], it still remains as a major complication of LC. It has been suggested that the LC related biliary injury is mostly attributed to the misidentification of the biliary duct anatomy [8, 9]. Since February 2007, we have tried a new modality to help identify biliary duct during LC by placing an optical fiber via endoscope in the common bile duct (CBD), the cold light from the fiber illuminates the cystic duct, CBD and the common hepatic duct, then titanium clips were applied to mark these anatomic structures and LC was performed under guidance of these clip marks. The function of clips is to mark the anatomic spots, becasue the illumination fiber can only illuminate the exposed Calot’s triangle but not the entire biliary duct. some patients were also performed cholangiography during LC if preoperative examinations showed CBD dilation. In this report, we summarize our experiences with these 16 patients underwent LC with optical fiber placement.

## Patients and Methods

### Patients

From February 2007 to June 2008, we performed LC with endoscopically placement of optical fiber in 16 randomly chosen patients with cholecystitis or/and cholelithiasis, six male patients, and 10 female patients, aged 48 - 85 years, average age 65.2 years. In these patients, 5 had elevated bilirubin, 6 had slight CBD dilation. The LC was performed in selective or non-selective basis.

### Materials

The laparoscopic instruments were purchased from Stryker Company (Kalamazoo, MI, USA). Duodenoscope was Olympus TJF-240 (Tokyo, Japan). The titanium clips, optical fiber and the cold light source were designed by the authors’ institute and manufactured by Hao Ke Medical Instruments Inc., Hang Zhou, China. The optical fiber was 150 cm long and 10 Fr in diameter, and it can be inserted into the biliary duct smoothly.

### Methods

LC was performed under general anesthesia and endotracheal intubation, artificial pneumoperitoneum was prepared and maintained pressure in 2 kpa. A port was made in the right umbilicus quadrant and was used for introduction of the laparoscope. After insertion of laparoscopy instruments, if abdominal adhesion was observed, dissection was made to expose the Carlot’s trangle, after this, the locations of gallbladder ampulla, cystic duct, CBD and common hepatic duct were preliminarily observed.

The patient was lying in supine, the operating bed was tilted left in 30 degree, a dental guard was used, then the duodenoscope was inserted, the procedure was the same as routine endoscopic retrograde cholangiopanceatography (ERCP). When the optical fiber was inserted to the duodenal Vater’s ampulla, the cold light source was turned on. The fiber was continually advanced to reach the proximal CBD or common hepatic duct, the light illuminating the Calot’s triangle would be observed, and slowly moving of the fiber would indentify the acurate locations of the CBD and common hepatic duct. At this moment, three 6-mm titanium clips were applied to the soft tissue surrounding the common hepatic duct, CBD, and the confluence of cystic duct with CBD, respectively; one titanium clip was applied to the surface of cystic duct near the infundibulum of gallbladder, thus the four clips showed a “T” shape. The cytic duct, CBD and common hepatic duct were acurrately identified and delineated in the operating field. The LC was performed under guidance of the clips locations. After the gallbladder was resected, the clips were removed. For the 5 patients with slight elevated blood bilirubin and 6 patients with slight CBD dilation, we also performed ERCP at the same time the optic fiber was inserted.

This study was approved by the IRB of our hospital, and patients’ consent was obtained prior to the procedure.

### Results

The clinical characteristics of the 16 patients and their outcomes were summarized in Table 1.

**Table 1:** Clinical Characteristics of the 16 Patients

Patient No.	Sex	Age (Y)	Disease	Operation Time (Min)	Intraoperative Complications	Post-operative Complications	Biliary Injury	Conversion to Open Surgery	Hospital Stay (d)
1	M	52	cholecystitis, cholelithiasis	74	N	N	N	N	7
2	F	68	cholecystitis, multiple cholelithiasis	62	N	N	N	N	6
3	M	76	cholecystitis, cholelithiasis, slight CBD dilation	70	N	N	N	N	9
4	M	82	cholecystitis, cholelithiasis, slight CBD dilation	58	N	N	N	N	6
5	M	48	cholecystitis, cholelithiasis	68	N	N	N	N	8
6	F	56	cholecystitis, multiple cholelithiasis	65	N	N	N	N	8
7	M	64	cholecystitis, cholelithiasis, slight CBD dilation	74	N	N	N	N	7
8	F	75	cholecystitis, multiple cholelithiasis,	95	N	Bile leak	N	N	11
9	F	85	cholecystitis, cholelithiasis, slight CBD dilation	76	N	N	N	N	7
10	F	65	cholecystitis, cholelithiasis	84	N	N	N	N	6
11	F	68	cholecystitis, cholelithiasis	90	N	Gallbladder fossa effusion	N	N	10
12	M	76	cholecystitis, cholelithiasis, slight CBD dilation	67	N	N	N	N	7
13	F	73	cholecystitis, cholelithiasis	84	N	N	N	N	8
14	F	56	cholecystitis, cholelithiasis, slight CBD dilation	96	N	N	N	N	6
15	F	51	cholecystitis, multiple cholelithiasis,	59	N	N	N	N	6
16	F	48	cholecystitis, multiple cholelithiasis,	67	N	N	N	N	5

M: male; F: female; CBD: common bile duct

In this series of 16 patients, optical fiber was placed successfully in 15 patients with sufficient light illumination, we failed in one patient due to the existence of peripapillary diverticulum, the fiber was unable to pass the ampulla of Vater. In the 15 successful patients, LC was performed uneventfully following the guidance of the clips identification. The total duration of endoscopic placement of optical fiber and clips was 17 - 35 minutes, mean 25 minutes. For those patients with signs of CBD dilation at pre-operative examinations, we performed cholangiography during the LC, and the cholangiography results showed slight biliary duct dilation in 8 patients, anomaly lower cystic duct opening in 2 patients. There were no complications related to LC or ERCP.

## Discussion

The incidence of BDI related to LC was reported 0.11-0.15% [8]. Generally, the BDI was derived from these three factors, anatomy, pathology and surgeon’s skill [10, 11].

### Anatomic factors

BDI may occur due to the misidentification of the anatomic structure of the Calot’s triangle. There exist anatomic variations of biliary ducts and blood vessels, including left and right hepatic duct, common hepatic duct, CBD, cystic duct, cystic artery, arteria hepatica propria, right and left hepatic artery, portal vein and its right and left branches. The confluence of cystic duct and common hepatic duct may have many variations with regards to the confluence angle, site and level. In addition, the accessory hepatic duct is the most important hepatic duct anatomic variation, this variation includes four types: (1) Confluence to the common hepatic duct via the Calot’s triangle; (2) Confluence to the CBD via the Calot’s triangle; (3) Confluence to the cystic duct directly; (4) Entry directly to the gallbladder through the liver tissue adjacent to the gallbladder, resulting in the gallbladder to hepatic duct. There also exist several anatomic variations for the cystic artery, with regards to its origin, route and numbers. In a small number of cases, the cystic artery might be originated from left hepatic artery, arteria hepatica propria, common hepatic artery, gastroduodenal artery, superior mesenteric artery and celiac artery.

### Pathological factors

In acute cholecystitis, swelling of Calot’s triangle usually occurs due to acute inflammation, especially when stones are incarcerated in the neck of gallbladder, the CBD is easily injured. During LC, if the CBD, common hepatic duct and cystic duct (neck of gallbladder) are not clearly recognized, the CBD and common hepatic duct are also easily injured.

### Technical factors

During the laparoscopic surgery, surgeons are unable to palpate the organs and tissues directly, the actual three dimensions of the organs do not appear as in the open surgery, also the operating view through the laparoscope is two dimensions, all these may contribute to the abdominal viscera injury [10].

To avoid the biliary injury, the following measures might be taken: (1) Ensure adequate skill training for the LC surgeons. (2) The retraction direction of the gallbladder neck should be vertical to the CBD, with appropriate retraction strength. During LC, the surgeons should observe the Calot’s triangle carefully from different angles and distances repeatedly. When the cystic duct is completely exposed, retraction should be removed. When necessary, the gallbladder bed in the portal area should be dissected partially first in order to expose the cystic duct, cystic blood vessels and their relations with the cystic duct. The titanium clips are applied only after the confluence of cystic duct and CBD is clearly recognized. (3) In the cases of cystic duct stones incarceration, swelling of Calot’s triangle, severe adhesion, misidentification of anatomical structure or variation, or uncontrollable bleeding, in all these conditions, the electric incision, electric cauterization or titanium clips should not be applied to dissect blindly, but conversion to open surgery should be immediately implemented. (4) Dissection of the cystic duct should start from the border of the gallbladder neck and cystic duct. The length of dissected cystic duct should not be too long, with 0.5 cm might be sufficient for clipping, and this is not too close to the CBD. (5) In the case of severe cystic duct swelling, 2 or 3 titanium clips might be applied in the proximal part, and make sure the clip near the CBD should not be too tight in order to avoid cystic duct necrosis and clip removal. (6) In case of selective LC, the intraoperative cholangiography (IOP) should be performed in the presence of the following conditions: preoperative jaundice; extra large cystic duct or CBD with small stones; adhesion of Calot’s triangle or unclear anatomical structure; and suspected biliary duct variation. (7) During LC, the gallbladder bed should be cauterized thoroughly for blocking the small aberrant bile ducts, thus to avoid bile leak. (8) If necessary, a drainage tube might be placed in the gallbladder bed, this way, the bile leak can be found timely.

It has been always an important issue to seek auxiliary approaches to identify biliary system during LC, especially the anatomy of Calot’s triangle, for avoidance of biliary injury. The commonly used initiatives include methylene blue cholangiography; intraoperative cholangiography (IOC); intraoperative ERCP; endoscopic placement of optical fiber in CBD as reported herein.

In a previous study reported 165 LC cases that had undergone IOC [12], the cystic duct was dissected along the gallbladder ampulla, the cystic duct was clipped near the ampulla, and a small oblique incision was made in the cystic duct 0.5 cm from the ampulla, then a catherter was inserted into the CBD for cholangiography. The results showed cystic variations in 22 cases, these include 8 cases of anomaly lower opening of cystic duct; one case of dual cystic ducts; 10 cases of extra shortness of cystic duct (less than 2 cm) in 10; 3 cases of opening to the right hepatic duct. Mirizzi syndrome was found in 12 cases, CBD stones in 8 cases. These findings imply that the IOC is useful for proper management of cystic duct and for ascertaining the indications of CBD exploration.

It is suggested that methylene blue IOC can effectively prevent biliary injury during LC [13, 14), this modality may help procure the biliary image directly and facilitate the dissection. However, when the gallbladder is filled with stones or the cystic duct is obstructed, methylene blue would not reach CBD thus not to be displayed. In addition, if white bile or purulent bile occurs, methylene blue cholangiography should not be used, for the methylene blue might leak and stain the Calot’s triangle, methylene blue can bind the visceral tissue tightly, particularly the dissected tissue, it could not be removed by flushing and renders dissection more difficult. The other disadvantage of methylene blue is that the stained image is vague, and there would be no adequate contrast between the stained biliary system and its peripheral tissue, this is even more significant when there is extra adipose tissue in the Calot’s triangle.

In another study, the Olsen cholangiocatheter was applied for cholangiography during LC [15]. However, because the trocar is fixed, the Olsen cholangiocatheter does not suit to the direction and angle of the cystic duct, thus failure of cannulation may occur; also this Olsen cholangiocatheter fixing forceps are quite expensive and can not be applied widely. Ying et al [16] reported a simplified guiding catheter for cholangiography, however, its procedures were complicated, time-consuming and hard to manipulate. It was reported that the direct cystic duct puncture other than the catheterizing cholangiography has yielded satisfactory outcome, with simplified procedures and uncomplicated instruments [17].

The light cholangiography (LCP) is the method that a light source is placed in the biliary duct. In a previous report with light cholangiogrphy in LC [18], surgeons made a small incision in the cystic duct near the neck of gallbladder, then the cold light source was turned on, a optical fiber was slowly advanced to the cystic duct through biliary cholangiography forceps, the light was seen in the cystic duct, when the optical fiber was slowly moved to the fundus of the gallbladder, the light was seen in the fundus of the gallbladder. Here, two points need to be addressed, firstly, the optical fiber should be advanced slowly, for the fiber is usually fine and less flexible, with rapid advancing, it may be mistakenly inserted into the peripheral tissue or even penetrate the whole gallbladder wall; secondly, for pre-evaluating the probability of insertion into the CBD, the fiber is advanced in various directions and moved back and forth, if the fiber is inserted from cystic duct to CBD, then the light may appear in the left or/and right hepatic duct; in the contrary, it is difficult to insert fiber from the CBD to cystic duct. Once the fiber is in the gallbladder and lightens the gallbladder wall, it is certain that the fiber is inserted via the cystic duct. It is suggested that the LCP is mainly suitable in the unclear biliary anatomy during LC. As an auxiliary approach, LCP may reduce the biliary injury in most cases, thus is recommended for wide use.

It has long been controversial whether the IOP should be routinely carried out. Most researchers from Western countries advocate that the IOP should be routinely applied in LC, some researchers advocate the cholangiography should be a selective approach. In our study, we placed optical fiber endoscopially in the CBD during LC, the light from the fiber illuminated and delineated the biliary system structures. For the patients with slight elevated blood bilirubin and slight dilated CBD, we also performed ERCP. Apparently, the LCP during LC has significant advantages, the images seen are live during LC, this may help surgeons make decisions for dissection, and it significantly increases the safety of the LC procedures. However, some technical points should be addressed for this modality. Firstly, the insertion of duodenoscope for placement of optical fiber is different from that of routine ERCP. Because the patient is in supine position with bronchial intubation, the dental guard is difficult to immobilize, consequently, this makes it more difficult to insert the duodenoscope through the esophagus. Surgeons should proceed carefully to prevent injury of pharynx. Secondly, because the tip of the optical fiber is not flexible, it is difficult to insert it through the Vater’s ampulla, this step should be performed by an experienced ERCP physician. Thirdly, due to the less flexibility of the optical fiber, the surgeons should advance it slowly in order not to penetrate the biliary duct wall, the endoscopist should cooperate with the LC surgeons thoroughly. Since the insertion of optical fiber was following the same procedure of routine ERCP, so the ERCP related complications may still possibly occur, although we did not encounter in our relatively small sample of patients.

In conclusion, we report a series of 16 cases underwent LC with light cholangiography, this modality can reduce unnecessary biliary duct exploration and reduce retaining of CBD stones, it contributes to the identification of normal and variation of the biliary duct anatomy, thus reduces the biliary injury related to LC. Further studies with larger samples are needed to fully assess the efficacy and feasibility of this modality.
